# Effectiveness of antimuscarinics and a beta-3 adrenoceptor agonist in patients with overactive bladder in a real-world setting

**DOI:** 10.1038/s41598-020-68170-4

**Published:** 2020-07-09

**Authors:** Chiung-Kun Huang, Chih-Chieh Lin, Alex Tong-Long Lin

**Affiliations:** 1 Department of Urology, Taipei Veterans General Hospital, No. 201, Section 2, Shih-Pai Road, Taipei, 11217 Taiwan, ROC; 2 Department of Urology, School of Medicine, National Yang-Ming University, Taipei, Taiwan, ROC; 3 Institute of Clinical Medicine, National Yang-Ming University, Taipei, Taiwan, ROC

**Keywords:** Diseases, Urology

## Abstract

Both antimuscarinics and beta-3 adrenoceptor agonists are generally used as first-line pharmacotherapy for overactive bladder (OAB). This study aimed to investigate the differences in clinical characteristics and manifestations between different medication groups using real-world data. In this retrospective study, we recruited all patients aged > 18 years diagnosed with OAB at our institute from March 2010 to December 2017. They were allocated into three groups, the antimuscarinics (group A), beta-3 adrenoceptor agonist (group B), and discontinued (group C) treatment groups, and they completed OAB symptom score and quality of life questionnaires before and after treatment. In addition, the Clinical Global Impression was recorded for treatment outcomes. A premedication urodynamic study was also applied. A total of 215 patients were analyzed (group A: 43, B: 35, C: 137). Group B was significantly older (mean age 77.4 years) than group A (69.2 years, *p* = 0.012) and group C (68.6 years, *p* = 0.001). However, there were no significant differences in sex or underlying diseases among the groups. Before treatment, there were no significant differences in the questionnaire results among all groups. The cystometric capacity of group A (mean ± SD, 257.3 ± 135.1 cm^3^) was significantly larger than that of group B (125.8 ± 46.0 cm^3^, *p* = 0.002) and group C (170.5 ± 99.2 cm^3^, *p* = 0.001). After treatment, there were no significant differences between group A and group B in any of the questionnaire scores; however, their scores were better than those of group C. The OAB patients who adhered to antimuscarinics tended to be younger and have larger cystometric bladder capacity in the urodynamic study. However, there were no significant differences in effectiveness between the patients who took antimuscarinics and those who took a beta-3 adrenoceptor agonist.

## Introduction

Overactive bladder (OAB) is defined as the presence of urinary urgency, usually accompanied by frequency and nocturia, with or without urgency urinary incontinence, in the absence of a urinary tract infection or other obvious pathology^1^. Updated treatment guidelines for OAB recommend that second-line treatment should consist of pharmacotherapy with oral antimuscarinic agents or the beta-3 adrenoceptor agonist mirabegron. Long-term pharmacotherapy is essential to control OAB symptoms and improve health outcomes^2,3^. Although antimuscarinic agents are effective for OAB control^4^, side effects, including dry mouth, constipation, blurred vision, fatigue and cognitive dysfunction, affect compliance and adherence to antimuscarinic treatment^5–7^. Several studies have reported that adherence to antimuscarinic treatment is low and decreases over time due to low efficacy and a high rate of adverse events^8^. Mirabegron is an alternative to antimuscarinic agents, especially in patients without improvements in overactive bladder symptoms such as urinary frequency, urgency and urgency incontinence after antimuscarinic treatment^9^. Although commonly reported adverse events of mirabegron treatment include hypertension (7.3%), nasopharyngitis (3.4%) and urinary tract infections (3%), mirabegron still has a more favorable tolerability rate in drug adherence and persistence than antimuscarinics^9,10^.

There is currently no consensus on which type of medication should be the first choice for patients with specific characteristics. Therefore, the aim of this research was to investigate the differences in clinical characteristics and manifestations between different medication groups using real-world data from our institute.

## Results

**Figure 1:**
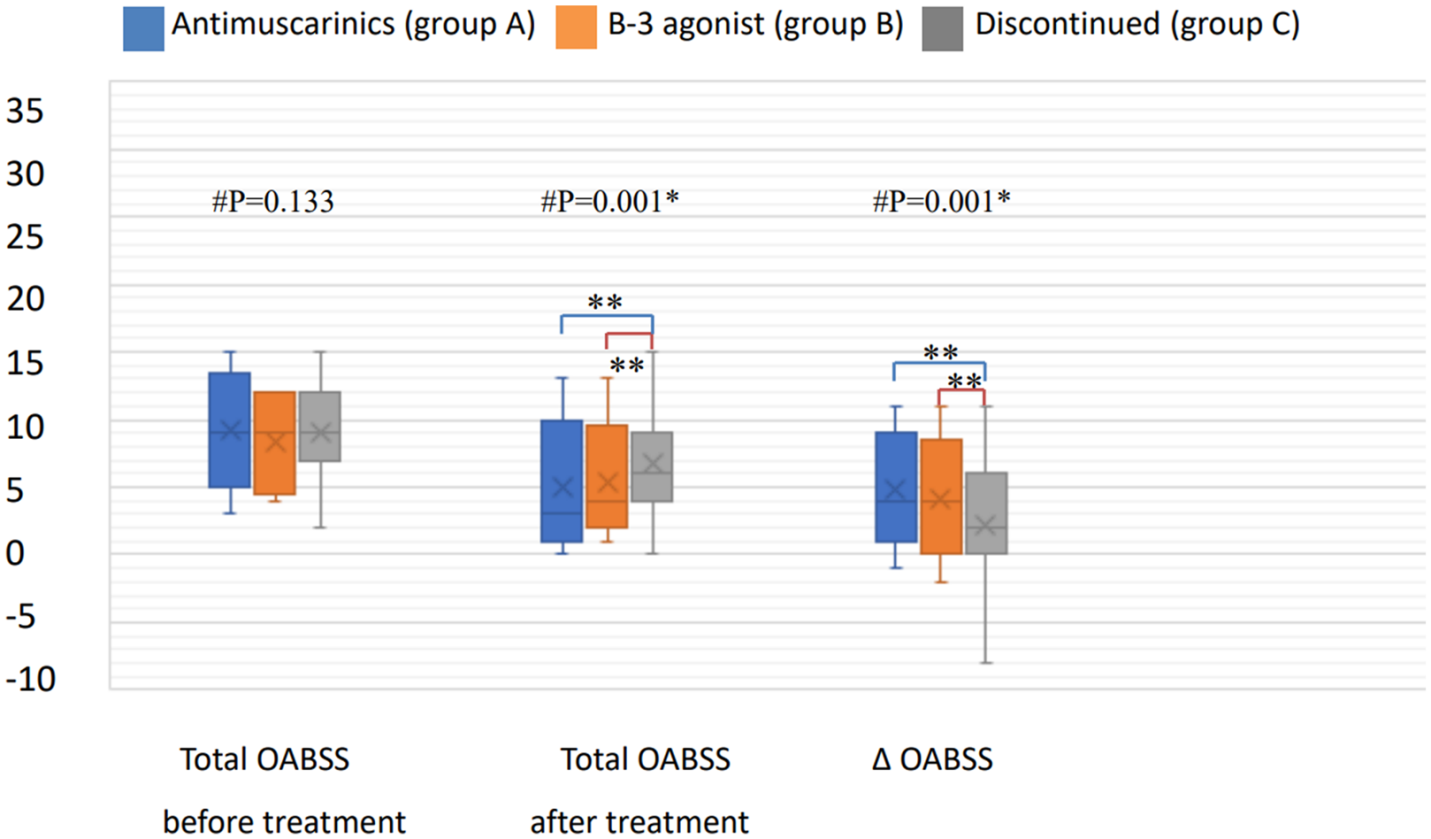
Comparison of OABSS questionnaire results in the three groups using the Mann–Whitney U test for statistical analysis. # Kruskal–Wallis test analysis among the three groups (*significant if *p* < 0.05). Post hoc multiple comparisons with Bonferroni correction, using the Mann–Whitney U test (**significant if *p* < 0.0167). No statistical significance between those receiving antimuscarinics (group A) and those receiving a beta-3 agonist (group B). Both groups A and B showed treatment efficacy compared to the discontinued group (group C).

**Figure 2:**
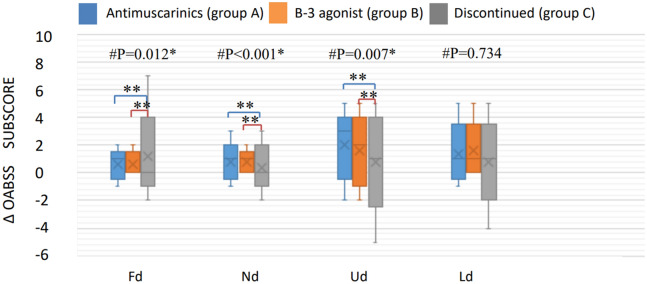
The differentiation of OABSS questionnaire subscores in the three groups. Fd: differentiation of daytime frequency score. Nd: differentiation of nighttime frequency score. Ud: Differentiation of urgency score. Ld: differentiation of urgency incontinence score. ^#^Kruskal–Wallis test analysis among the three groups (*significant if *p* < 0.05). Post hoc multiple comparison with Bonferroni correction, using the Mann–Whitney U test (**significant if *p* < 0.0167). No statistically significant difference between those receiving antimuscarinics (group A) and those receiving a beta-3 agonist (group B). Both groups A and B showed treatment efficacy compared to the discontinued group (group C).

**Figure 3:**
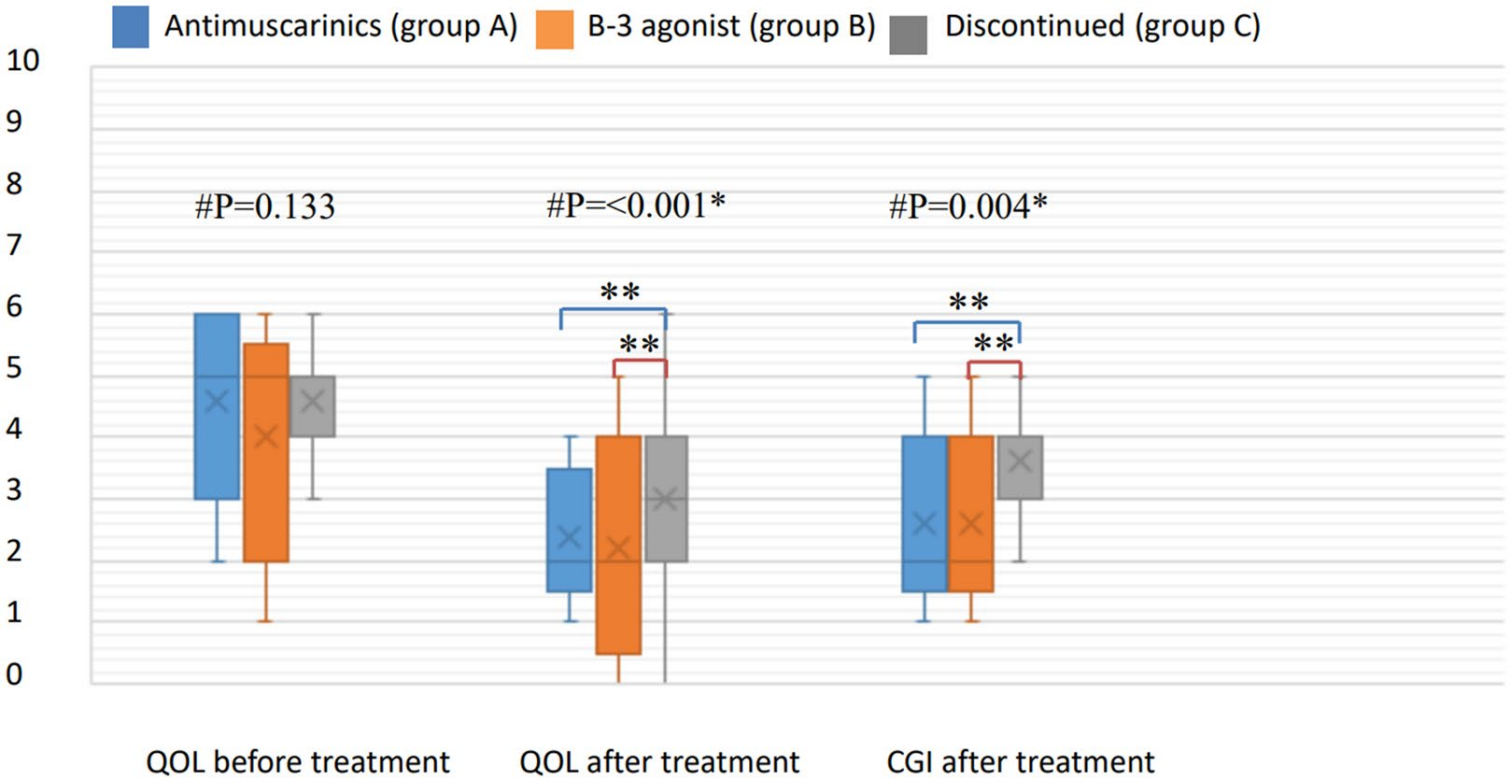
Comparison of QoL of the patients and CGI scores in each treatment group. ^#^Kruskal–Wallis test analysis among the three groups (**significant if *p* < 0.05). Post hoc multiple comparisons with Bonferroni correction, using the Mann–Whitney U test (**significant if *p* < 0.0167). No statistical significance between those receiving antimuscarinics (group A) and those receiving a beta-3 agonist (group B). Both groups A and B showed treatment efficacy compared to the discontinued group (group C).

Of the 215 patients included in this study, 43 (20%) were classified into group A, 35 (16.2%) into group B, and 137 (63.8%) into group C. There were no significant differences in sex or underlying diseases among the three groups (Table 1).However, the mean age of group B (77.4 ± 12.6 years, range 49 to 95 years) was significantly older than that of group A (69.3 ± 15.2 years, range 31–91 years, *p* = 0.012) and group C (68.7 ± 14.1 years, range 20–90 years, *p* = 0.001). In urodynamic studies, there were no significant differences in any of the urodynamic parameters among the three groups except for cystometric (CMG) capacity. Group A had a significantly larger CMG capacity (mean ± SD, 257.3 ± 135.1 cm^3^, range 89–497 cm^3^) than group B (125.8 ± 46.0 cm^3^, range 76–189 cm^3^, *p* = 0.002) and group C (170.5 ± 99.2 cm^3^, range 86–425 cm^3^,* p* = 0.001) (Table 2). Medication group A consisted of antimuscarinic-naïve individuals (n = 35) and those who had discontinued mirabegron treatment (n = 8); the CMG capacity of each subgroup was 260.9 ± 119.2 cm^3^ (n = 35) and 243.2 ± 102.6 cm^3^s(n = 8), *p* = 0.7, respectively. Group B comprised mirabegron-naïve patients (n = 30) and those who had discontinued antimuscarinic treatment (n = 5); the CMG capacity of each subgroup was 123.1 ± 43.6 (n = 30) and 142 ± 53.8 (n = 5), *p* = 0.87, respectively. There was no significant difference in the mean duration of treatment between groups A and B (*m*_*A*_ = 527.0 days, range 28–1980 days and. *m*_*B*_ = 516.4 days, range 112–1925 days; *p* = 0.9). There were no significant differences in questionnaire scores, including the OABSS and QoL, among the three groups before treatment; however, significant differences were noted in the OABSS in group A (median 4, range − 1 to 11) and group B (median 4, range − 2 to 11) after treatment. Compared to group C (median 2, range − 8 to 11), the OABSS total in both groups A and B significantly improved after medication (*p* = 0.002 and *p* = 0.006, respectively) (Fig. 1). Both treatment groups showed better responses on the QoL and the CGI questionnaires after treatment (Fig. 2), showing that both antimuscarinics and beta-3 adrenoceptor agonists were effective medications. There was no significant difference between group A and group B in CGI after treatment (*p* = 0.135). We further analyzed differences in subscores of every OABSS scale before and after treatment. The results also showed no significant differences between group A and group B in daytime frequency score, nighttime frequency score, urgency score, or urge incontinence score (Fig. 3).

**Table 1:** Comparison of demographic and clinical characteristics of the patients in each treatment group.

	Antimuscarinics (A) (n = 43)	Beta-3 agonist (B) (n = 35)	Discontinuation(C) (n = 137)	*P*
Sex(M/F)	27/16	24/11	90/47	0.866
Age (years ± SD)	69.3 ± 15.2	77.4 ± 12.6	68.7 ± 14.1	0.005
Hypertension	48.8%	45.7%	46.7%	0.957
Diabetes mellitus	18.6%	25.7%	19.3%	0.669
Hyperlipidemia	20.9%	22.9%	16.4%	0.607
COPD	4.7%	5.7%	2.3%	0.520

*COPD* chronic obstructive pulmonary disease.

Pearson’s chi-squared (*significant if *p* < 0.05).

**Table 2:** Comparison of urodynamic parameters of the patients in each treatment group by using ANOVA and Kruskal–Wallis test.

	Antimuscarinics (n = 43)	Beta-3 agonist (n = 35)	Discontinued (n = 137)	*P*
First desire capacity (ml)	127.3 ± 82.1	85.6 ± 32.1	105.0 ± 72.0	0.277
CMG capacity (ml)	257.3 ± 135.1	125.8 ± 46.0	170.5 ± 99.2	0.01*
Pdet at Qmax (cm H_2_O)	48.2 ± 21.8	52.9 ± 33.9	50.9 ± 26.2	0.877
Qmax (ml/s)	19.0 ± 12.6	13.1 ± 7.6	15.4 ± 10.2	0.079
PVR (ml)	54.6 ± 106.4	25.7 ± 31.5	39.1 ± 59.6	0.442

CMG capacity, group comparison (antimuscarinics vs. beta-3 agonist, ^#^*p* = 0.002).

*PVR* postvoid residual.

Kruskal–Wallis test (*significant if *p* < 0.05). Post hoc multiple comparisons with Bonferroni correction using the LSD test to compare each group (^#^significant if *p* < 0.0167).

The discontinuation factors were also collected from telephone interviews or medical records. They were identified as having poor efficacy (47.4%), adverse events (19.7%), preferred lifestyle modifications (15.3%), feared deterioration of renal function owing to long-term medication (11.5%) and diaper use (5.8%). Adverse effects comprised intolerable dry mouth (57.7%), constipation (30.8%), urinary retention (7.7%) and blurred vision (3.8%).

## Discussion

In this study, we used demographic data, urodynamic tests and questionnaires (subscores of the OABSS) before pharmacotherapy to analyze the characteristics of patients who received antimuscarinics, those who received a beta-3 adrenoceptor agonist, and those who discontinued treatment. The patients who received antimuscarinics had a significantly larger CMG capacity and were significantly younger than those who received the beta-3 adrenoceptor agonist mirabegron. In systemic reviews^3,11^, antimuscarinics have been shown to act mainly during the urinary storage phase and to decrease the activity of afferent bladder nerves, resulting in decreased urgency and increased bladder capacity. One prospective study enrolling 122 women who received 12 weeks of solifenacin treatment reported higher antimuscarinic persistence and retreatment rates when the patients had frequent nocturia episodes, a suboptimal response, and small bladder capacity^17^. In our series, the patients with a larger bladder capacity before treatment showed better persistence and adherence to antimuscarinic treatment. A larger bladder capacity may decrease the number of daily voiding episodes as antimuscarinics decrease the frequency of bladder spasms. On the other hand, mirabegron has been shown to improve storage function and increase maximum cystometric capacity in real-world studies^12,13^. In addition, beta-3 adrenergic receptor agonists (FK175, CL316243) have been shown to significantly increase bladder capacity and prolong micturition intervals without affecting voiding pressure or postvoid residual volume^14^. Therefore, patients with a small bladder capacity may be more suitable for mirabegron treatment.

In this study, the younger patients seemed to have better adherence to antimuscarinic agents. Yoshida et al. demonstrated a significant positive correlation between age and purinergic neurotransmission and a significant negative correlation between age and cholinergic neurotransmission in neurogenic contractions of human bladder smooth muscle^15^. They concluded that reduced cholinergic neurotransmission resulted in a poorer response to antimuscarinic treatment. Chapple et al. reported that mirabegron also showed better persistence (time to discontinuation, 12-month persistence) and adherence (medication possession rate) rates than antimuscarinic agents in elderly patients^16^.

In our series, mirabegron was prescribed to patients who responded poorly to antimuscarinic monotherapy or to men with significant storage/OAB symptoms^9^, as in Chapple’s study. Head-to-head comparisons were conducted, and telephone interviews were performed at the endpoint of this research. OAB is a bothersome symptom when the daily QoL is affected. The OABSS, QoL and CGI questionnaires are validated tools to assess treatment efficacy and health-related QoL. We also analyzed the subscores of the OABSS and compared changes in both storage and voiding symptoms, which not only standardized the assessments but also revealed the treatment efficacy. Kelleher et al. demonstrated that both antimuscarinics and mirabegron are effective for OAB and that the main issue in the discontinuation of OAB treatment is tolerability. Mirabegron was better tolerated than antimuscarinics regarding some side effects^17^. Our results also showed that patients who kept antimuscarinics and the beta-3 adrenoceptor agonist showed better treatment outcome compared to discontinued group. Both agents showed no significant differences in storage or voiding subscores between the two groups. However, in the current study, we investigated the predictive factors, which will provide us with information on better adherence to OAB treatment before initiating OAB treatment. In particular, we analyzed urodynamic parameters before OAB treatment.

There are several limitations to this study. We excluded patients who received combination therapy with mirabegron plus an antimuscarinic agent, and we did not consider escalations in the dose of pharmacotherapy. A voiding diary is also a reliable tool to objectively measure mean voided volume, daytime and nighttime frequency, and the frequency of incontinence episodes. Bladder outlet obstruction may also play an important role in OAB in male patients. Lower-abdominal sonograms to evaluate the prostate and bladder wall may provide meaningful parameters by quantifying the thickness of the bladder wall or the intravesical prostatic protrusion (IPP) grade. The incidence of IPP has been reported to be significantly higher in patients with detrusor instability than in patients with a stable bladder (53% vs. 13%, *p* < 0.01)^10^. Male OAB has also been correlated with IPP grade^18^. However, voiding diaries and image surveys were not applied in this study. In addition, this was a retrospective and single-center study. Further studies are needed to validate our results.

## Conclusion

The OAB patients who adhered to antimuscarinics (group A) tended to have larger cystometric bladder capacity in the urodynamic study. Additionally, patients in beta-3 adrenoceptor agonist group (group B) were significantly older than other groups. Patients who kept antimuscarinics and the beta-3 adrenoceptor agonist showed better treatment outcome compared to discontinued group. There was no significant difference in the treatment outcome between these two pharmacotherapies.

## Materials and methods

**Figure 4:**
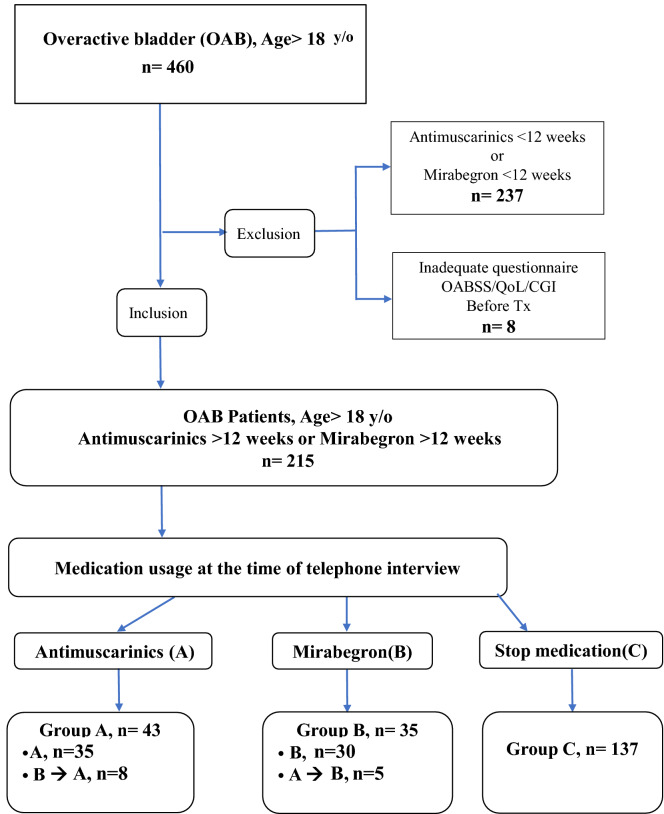
The inclusion criteria for the enrolled OAB patients assigned to three different pharmacotherapy groups. Inclusion criteria for overactive bladder (OAB). *Pharmacotherapy: either antimuscarinics or mirabegron, total pharmacotherapy duration of more than 4 weeks despite switching medication. The patients were divided into three groups by latest, longest and persistent medication determined by phone inquiry in December 2017 (Group A: antimuscarinics; Group B: mirabegron; Group C: discontinued).

In this retrospective single-center study, all OAB patients aged more than 18 years old were included after signing the informed consent form. Every patient met the criteria for the diagnosis of OAB proposed by the International Continence Society in 2002. Patients with neurogenic bladder and cancer of the genitourinary tract were excluded. All methods were carried out in accordance with relevant guidelines and regulations. All experimental protocols were approved by the ethical committee of our institute (IRB No.201001032IC). All medications were randomly prescribed by Lin A.T.L. and Lin C.C. All patients with OAB received pharmacotherapy with antimuscarinics or a beta-3 adrenoceptor agonist (mirabegron) for more than 12 weeks at our institute from March 2010 to December 2017. The telephone interviews were conducted by Huang C.K. at the endpoint of this research (December 2017). Participants were categorized into three groups according to final treatment: the antimuscarinics (group A, including oxybutynin ER 5 mg, solifenacin ER 5 mg, and tolterodine ER 4 mg), beta-3 adrenoceptor agonist (group B, mirabegron 25 mg, some patients received antimuscarinics first and then switched to mirabegron due to adverse effects),and discontinued (group C) groups. The patients who switched their medication from antimuscarinics or beta-3 adrenoceptor agonist to other medications were allocated into group A or B according to the final and longest duration of treatment with that agent, as long as the duration was more than 12 weeks. Patients in the discontinued group (group C) had ceased medication at the time of telephone inquiry and had received either antimuscarinics or a beta-3 adrenoceptor agonist (Fig. 4).

Urodynamic studies and questionnaires, including the Overactive Bladder Symptom Score (OABSS) and global improvement scale of the Clinical Global Impression (CGI), have been shown to be important and reliable tools in previous studies to assess treatment outcomes in OAB patients^19,20^. Therefore, all patients received questionnaires, including the OABSS and Quality of Life (QoL) questionnaires, before and after taking medication. The global improvement scale of the CGI and differences in OABSS subscores were recorded to evaluate treatment outcomes. Cystometry was performed before treatment. In the statistical analysis, Pearson's chi-squared test was used to evaluate the incidence of underlying diseases among the three groups. All urodynamic parameters and questionnaires were statistically analyzed with ANOVA and the Kruskal–Wallis test. Concerning the comparatively small sample size, ANOVA was applied if the dependent variables are normally distributed whereas Kruskal–Wallis test was applied if they are not. Post hoc comparison with LSD correction and Mann–Whitney tests using a Bonferroni-adjusted alpha level (significant if *p* < 0.0167) were performed respectively.
